# A Na^+^/Ca^2+^ exchanger of the olive pathogen *Pseudomonas savastanoi* pv. *savastanoi* is critical for its virulence

**DOI:** 10.1111/mpp.12787

**Published:** 2019-03-26

**Authors:** Chiaraluce Moretti, Simone Trabalza, Letizia Granieri, Eloy Caballo‐Ponce, Giulia Devescovi, Alberto Marco Del Pino, Cayo Ramos, Vittorio Venturi, Harrold A. van den Burg, Roberto Buonaurio, Carlo Alberto Palmerini

**Affiliations:** ^1^ Department of Agricultural, Food and Environmental Science University of Perugia Borgo XX Giugno 74, Perugia 06121 Italy; ^2^ Instituto de Hortofruticultura Subtropical y Mediterránea La Mayora Universidad de Málaga‐Consejo Superior de Investigaciones Científicas (IHSM‐UMACSIC) Área de Genética Málaga Spain; ^3^ Bacteriology Group, International Centre for Genetic Engineering and Biotechnology Trieste Italy; ^4^ Molecular Plant Pathology, Swammerdam Institute for Life Sciences (SILS) University of Amsterdam Amsterdam Netherlands

**Keywords:** calcium, β‐galactosidase assay, host detection, Na^+^/Ca^2+^ exchanger, olive knot disease, pathogenicity factor, *Pseudomonas savastanoi* pv. *savastanoi*

## Abstract

In a number of compatible plant‐bacterium interactions, a rise in apoplastic Ca^2+^ levels is observed, suggesting that Ca^2+^ represents an important environmental clue, as reported for bacteria infecting mammalians. We demonstrate that Ca^2+ ^entry in *Pseudomonas savastanoi* pv. *savastanoi* (*Psav*) strain DAPP‐PG 722 is mediated by a Na^+^/Ca^2+^ exchanger critical for virulence. Using the fluorescent Ca^2+^ probe Fura 2‐AM, we demonstrate that Ca^2+^ enters *Psav* cells foremost when they experience low levels of energy, a situation mimicking the apoplastic fluid. In fact, Ca^2+^ entry was suppressed in the presence of high concentrations of glucose, fructose, sucrose or adenosine triphosphate (ATP). Since Ca^2+^ entry was inhibited by nifedipine and LiCl, we conclude that the channel for Ca^2+^ entry is a Na^+^/Ca^2+^ exchanger. *In silico* analysis of the *Psav* DAPP‐PG 722 genome revealed the presence of a single gene coding for a Na^+^/Ca^2+^ exchanger (*cneA*), which is a widely conserved and ancestral gene within the *P.* *syringae* complex based on gene phylogeny. Mutation of *cneA* compromised not only Ca^2+ ^entry, but also compromised the Hypersensitive response (HR) in tobacco leaves and blocked the ability to induce knots in olive stems. The expression of both pathogenicity (*hrpL*, *hrpA* and *iaaM*) and virulence (*ptz*) genes was reduced in this *Psav*‐*cneA* mutant. Complementation of the *Psav*‐*cneA* mutation restored both Ca^2+^ entry and pathogenicity in olive plants, but failed to restore the HR in tobacco leaves. In conclusion, Ca^2+^ entry acts as a ‘host signal’ that allows and promotes *Psav* pathogenicity on olive plants.

## Introduction

Cytosolic calcium (Ca^2+^) has essential functions in eukaryotic signalling as secondary messenger. The cytosolic Ca^2+ ^levels are influenced by the difference in its intracellular‐to‐extracellular concentration (Berridge *et al*., 2000; Bhosale *et al*., 2015; Islam, 2012;). In particular in mammals, Ca^2+^ signalling is well understood with a central role in nearly all the known cellular processes ranging from egg‐cell fertilization to programmed cell death (Brini *et al*., 2013; Rajagopal and Ponnusamy, 2017), impacting gene expression levels, heart and muscle contraction, neurotransmission and synaptic plasticity, secretion of hormones and their action, blood coagulation and other motility processes, to diverse metabolic pathways involved in the generation of cell fuels (Sharma *et al*., 2017). Furthermore, Ca^2+^ acts both as a messenger and cofactor to coordinate many intracellular signalling pathways (Rajagopal and Ponnusamy, 2017). Noteworthy, it can already activate different cellular responses only by differences in the amplitude, frequency and duration of the intracellular Ca^2+ ^concentration (Rajagopal and Ponnusamy, 2017). Located predominantly in the extracellular environment, Ca^2+^ entry relies in animals on membrane depolarization resulting from action potentials, where it then can perform its regulatory functions. In these eukaryotes, most ion channels as well as transporters, pumps, binding proteins and L‐type voltage‐dependent calcium channels have the capacity to transport Ca^2+^ across the depolarized membrane (Cai and Lytton, 2004; Carafoli, 1987; Norris *et al*., 1996; Tsien and Tsien, 1990). In plants, Ca^2+^ is present in high concentrations in the apoplast (*i.e.* intercellular spaces and xylem) (Fishman *et al*., 2018) and Ca^2+^ influx can for example, activate plant defences (Aslam *et al*., 2008). Furthermore, Ca^2+^ signalling plays an essential role in pollen tube elongation, seed germination, hyperosmotic and oxidative stresses (Sanders *et al*., 1999; White and Broadley, 2003).

Although the molecular mechanisms that cause the cytosolic fluctuations of Ca^2+^ levels are well understood for eukaryotic cells, much remains to be discovered for prokaryotes. Nevertheless, there is a growing amount of evidence that Ca^2+^ also plays an important regulatory role in the physiology of prokaryotes (Fishman *et al*., 2018). However, due to their small cell size, the selective permeability of their cell walls and cell membrane and the toxicity of many chelators used in these Ca^2+^ studies, it remains complex to monitor Ca^2+^ concentrations inside bacterial cells, which is nevertheless indispensable to increase our understanding of the connection between Ca^2+^ influx and other cellular processes. The use of the Ca^2+^ reporters aequorin (Watkins *et al*., 1995) and Fura 2 (1‐[2‐(5‐carboxyoxazol‐2‐yl)‐6‐amino‐benzofuran‐5‐oxy]‐2‐(2′‐amino‐5′‐methylphenoxy) ethan‐*N*,*N*,*N*′,*N*′‐tetraacetic acid) (Gangola and Rosen, 1987; Tisa and Adler, 1995) revealed that variations in cytosolic Ca^2+^ levels also regulate many important bacterial cellular processes. For example, Ca^2+^ acts in bacteria, including plant pathogenic bacteria, as a versatile intracellular messenger involved in the maintenance of cell structure (Domínguez *et al*., 2015), motility (Cruz *et al*., 2012; Fishman *et al*., 2018; Gode‐Potratz *et al*., 2010; Guragain *et al*., 2013; Parker *et al*., 2015; Tisa and Adler, 1995), cell division (Domínguez *et al*., 2015), gene expression (Domínguez *et al*., 2015), type III secretion (Dasgupta *et al*., 2006; DeBord *et al*., 2003; Fishman *et al*., 2018; Gode‐Potratz *et al*., 2010), exopolysaccharide production (Kierek and Watnick, 2003; Kim *et al*., 1999; Patrauchan *et al*., 2007), iron scavenging (Domínguez *et al*., 2015; Patrauchan *et al*., 2007), quorum sensing (Werthén and Lundgren, 2001), biofilm formation (Cruz *et al*., 2012; Das *et al*., 2014; Parker Jennifer *et al*., 2016; Patrauchan *et al*., 2005; Rinaudi *et al*., 2006; Sarkisova *et al*., 2005; Zhou *et al*., 2013) or biofilm suppression (Bilecen and Yildiz, 2009; Shukla and Rao, 2013). Furthermore, Ca^2+^ appears to determine the virulence of the facultative human pathogen *Pseudomonas aeruginosa* (Guragain *et al*., 2016; Patrauchan *et al*., 2007; Sarkisova *et al*., 2014) and of all species of *Yersinia* (Mekalanos, 1992). Hardly any information is available on the role of Ca^2+^ for virulence of phytopathogenic bacteria. It was recently demonstrated that a two‐component system induced by Ca^2+^ controls virulence of the model plant pathogen *Pseudomonas syringae* pv. *tomato* DC3000 (Fishman *et al*., 2018). Here, we report on the role of Ca^2+^ for virulence of *Pseudomonas savastanoi* pv. *savastanoi* (referred to as *Psav*), the causal agent of olive knot disease.

Olive knot disease is characterized by knots or gall outgrowths on mainly twigs and young plant branches, while leaf and fruit infections are rare and only develop during wet summers. *Psav* survives as an epiphyte in the phyllosphere penetrating its host through wounds (Lavermicocca and Surico, 1987). Once inside host plants, the bacterium colonizes the apoplast and due to its ability to secrete the plant hormones indole‐3‐acetic acid (IAA) and cytokinins, it stimulates olive cells to produce new tissue giving rise to knot development and tissue overgrowth (Glass and Kosuge, 1988; Powell and Morris, 1986; Quesada *et al*., 2012; Ramos *et al*., 2012; Rodríguez‐Moreno *et al*., 2008; Surico *et al*., 1985; Temsah *et al*., 2008). The switch from an epiphytic to endophytic (apoplastic) life style is an abrupt transition for the bacterium that requires: (i) a remarkable adaptation to an environment that is extremely different in pH, osmotic pressure, carbon sources and oxygen availability, (ii) the ability to suppress basal and induced plant defences (Rico *et al*., 2009). Although the bacterial signals (*e.g.* flagellin, elongation factors) that the plant perceives through specific receptors and via which it activates plant immunity have been extensively studied in the last decades (Buonaurio, 2008; Chisholm *et al*., 2006; Dangl *et al*., 2013; Jones and Dangl, 2006; Silva *et al*., 2018), little is known on the molecular signals that the phytopathogenic bacteria perceive during this transition to the apoplast. We here reveal that Ca^2+^ influx in *Psav* is stimulated by low energy situations and that it requires a Na^+^/Ca^2+^ exchanger that is essential for *Psav *virulence on olive plants.

## Results

### Ca^2+^ entry in *Psav* cells is promoted under starvation conditions and is not influenced by exogenous indole‐3‐acetic acid

**Figure 1:**
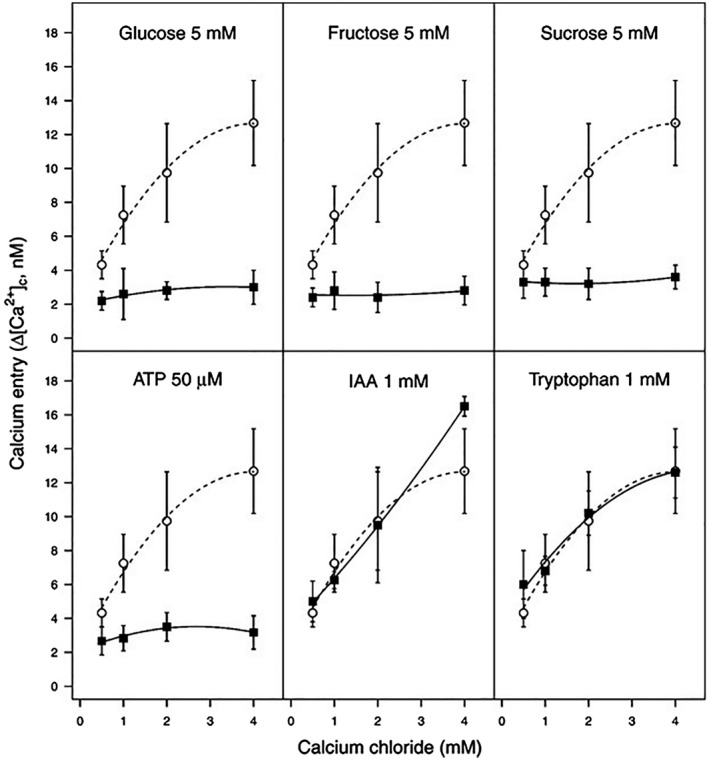
Increase of cytosolic Ca^2+^ levels in *Pseudomonas savastanoi *pv.* savastanoi *DAPP‐PG 722 cells incubated in HBSS medium alone (basal conditions; open circles) or in the presence of glucose, fructose, sucrose, ATP, indole 3 acetic acid (IAA) or tryptophan (closed squares) over a concentration range extracellular calcium chloride. Each point is the mean of 10 independent experiments ± SE.

Our understanding of molecular signalling in the early phases of plant bacterial infection is limited, while this early signalling largely defines the onset of bacterial disease. Since Ca^2+^ is a well‐known signalling molecule in plants and animals, we here investigated the role of Ca^2+^ signalling for a bacterial pathogen. We chose the olive – *Psav* pathosystem and used a biochemical approach to study if Ca^2+^ signalling is important for pathogenicity and virulence. First, we assessed if the cytosolic Ca^2+^ concentration of *Psav* is influenced by external Ca^2+^. To this end, we measured in *Psav* DAPP‐PG 722 cells under basal conditions (*i.e.* Hanks’ Balanced Salt Solution, HBSS buffer) whether an increase in external Ca^2+^ resulted in an increase in the cytosolic Ca^2+^ concentrations in *Psav*. We find that the cytosolic Ca^2+^ concentrations rapidly increase in response to external Ca^2+^ concentration in the medium (Fig. 1). This trend was suppressed when different carbon sources (glucose, fructose or sucrose) or ATP were added in a combination with Ca^2+^ (Fig. 1). Since IAA is produced by *Psav* to stimulate plant cell proliferation and knot formation, we also investigated whether IAA or its precursor (L‐tryptophan) influences Ca^2+^ entry. However, addition of IAA or L‐tryptophan to the incubation buffer did not significantly alter Ca^2+^ entry in *Psav* DAPP‐PG 722 cells (Fig. 1). Combined, these data suggest that *Psav *actively controls Ca^2+^ entry rather than that this Ca^2+^ influx represents a passive process.

### Ca^2+^ entry in *Psav* cells is mediated by the Na^+^/Ca^2+^ exchanger CneA

**Figure 2:**
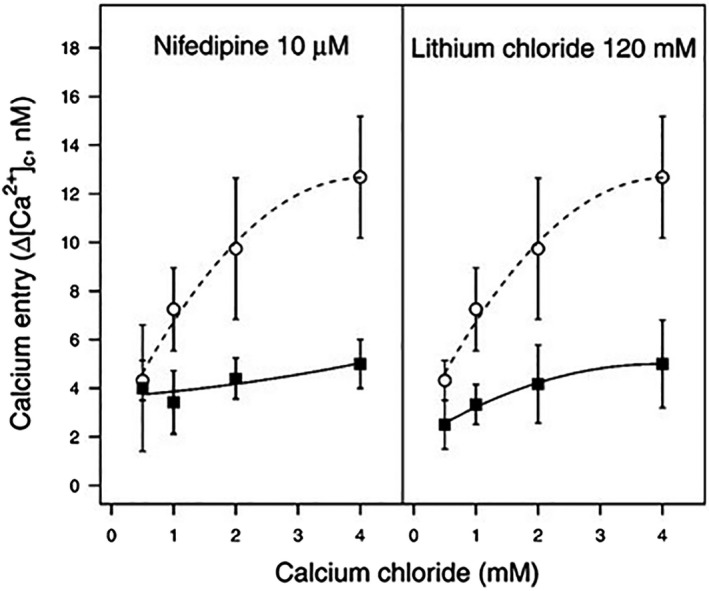
Increase of cytosolic Ca^2+^ levels in *Pseudomonas savastanoi *pv.* savastanoi *DAPP‐PG 722 cells pre‐treated with nifedipine, Lithium chloride (squares) and the negative control (circles) after which the cells were incubated in HBSS medium at different concentrations of extracellular calcium chloride. Each point is the mean of 10 independent experiments ± SE.

To determine if Ca^2+^ entry depends on an ion channel, *Psav* DAPP‐PG 722 cells were pre‐treated with nifedipine, an inhibitor of the L‐voltage channels responsible for the entry of the extracellular Ca^2+^ in mammals (Sorkin *et al*., 1985) or LiCl that, in substitution of Na^+^ in the buffer, inhibits Na^+^/Ca^2+^ exchangers (Yanagita *et al*., 2007). Since Ca^2+^ entry was inhibited by both nifedipine and LiCl (Fig. 2), we conclude that a Na^+^/Ca^2+^ exchanger is potentially involved in the entry of extracellular Ca^2+^ in *Psav*. *In silico* analysis of the genome of *Psav* DAPP‐PG 722 (Moretti *et al*., 2014) revealed the presence of a single gene coding for a Na^+^/Ca^2+^ exchanger (here designated as *cneA*; MK408668), which belongs to the ChaA antiporter superfamily (Shijuku *et al*., 2002). This *cneA* gene encodes for a protein (CneA) that encompasses the PRK10599, caca2 and Na^+^/Ca^2+^ exchanger protein domains (Marchler‐Bauer *et al*., 2017). In the genome of *Psav* DAPP‐PG 722, two genes are located directly upstream of *cneA* gene, which encode for a guanine deaminase and hydroxydechloroatrazine ethylaminohydrolase, while downstream we find a gene encoding an iron(III) dicitrate transport system. A comparative phylogenetic analysis of the nucleotide sequences of the *cneA* gene was performed using the Geneious resource (Kearse *et al*., 2012). Sequences of this gene were retrieved from a series of strains that belong to the seven primary (monophyletic) phylogroups (PGs) described for the *P. syringae* complex. Homologs of the *cneA* gene were found to be widely distributed across the *P. syringae* complex. However, the branching of the *cneA* gene tree was not fully consistent with the previously reported phylogeny of the *P. syringae* species complex that is based on a multilocus sequence analysis (MLSA) of housekeeping genes (Baltrus *et al*., 2017). This suggests that the *cneA* gene has undergone horizontal gene transfer between species in this bacterial complex. For example, although PG2, PG3 and PG6 are equally distributed in a common branch in both the *cneA* and the MLSA phylogeny, some PG3 pathovars (*e.g. P. syringae* pathovars *cunninghamiae*, *castaneae*, *photiniae* and *myricae*, amongst others) have a different position in the *cneA* gene tree than in the MLSA tree (Fig. S1).

### A *Psav*‐*cneA* mutant is inhibited in Ca^2+^ entry and is unable to induce both the hypersensitive response (HR) in *Nicotiana tabacum* and formation of knots on olive plants

**Figure 3:**
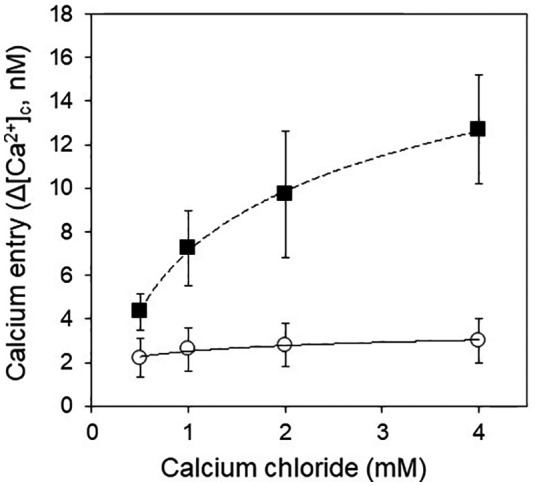
Increase of cytosolic Ca^2+^ levels in *Pseudomonas savastanoi *pv.* savastanoi* wild type (closed squares) and *Psav*‐*cneA* mutant (open circles) cells incubated in HBSS medium at different concentrations of extracellular calcium chloride. Each point is the mean of 10 independent experiments ± SE.

In order to investigate the role of the *Psav*‐*cneA* gene in Ca^2+^ entry, a *Psav* DAPP‐PG 722 *cneA* mutant was constructed and its ability to transport Ca^2+^ into the cytosol was tested in comparison to the wild‐type strain. For this purpose, *Psav* cells were incubated in basal conditions or in the presence of glucose. The uptake of Ca^2+^ was strongly impaired in the mutant cells incubated under basal condition (Fig. 3). It is worth mentioning that the *in vitro *growth rate of *Psav*‐*cneA* mutant cells was identical to that of the *Psav* wild‐type strain in KB medium (Likelihood ratio test, *P‐*value = 0.85; Fig. S2).

**Figure 4:**
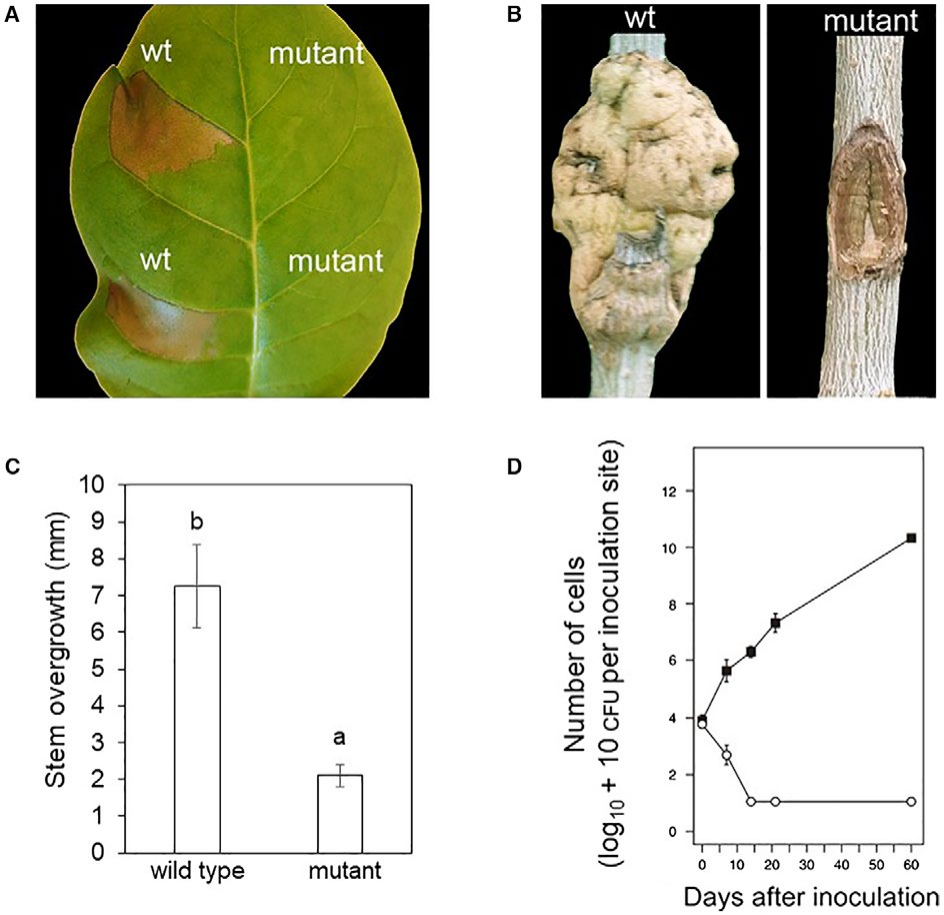
Role of the calcium exchanger on pathogenicity and virulence of* Pseudomonas savastanoi *pv.* savastanoi* (*Psav*) using a wild type (wt) isolate and the *Psav*‐*cneA* mutant. (A) HR in tobacco (cv. Havana 425) leaves, 24 h after the infiltration of *Psav* wt or *Psav*‐*cneA* mutant. (B) Knot formation in 1‐year‐old olive (cv. Frantoio) stems inoculated with *Psav* wt or *Psav*‐*cneA* mutant. (C) Knot thickness measured in *Psav* wt and *Psav*‐*cneA* mutant inoculated olive plants. Each column represent the mean of four replicates ± S.E. Columns capped with different letter are significantly different (*P* = 0.01) according to the Fisher’s test. (D) Population dynamics of *Psav* wt (closed squares) and *Psav*‐*cneA* mutant (open circles) inoculated in olive plants. Each point is the mean of four replicates ± SE.

To examine whether Ca^2+^ entry was involved in *Psav* pathogenicity and virulence, both the *Psav* DAPP‐PG 722 wild type and *Psav*‐*cneA* mutant were: (i) infiltrated in the non‐host tobacco, (ii) inoculated on 1‐year‐old wounded olive plants. When infiltrated in tobacco leaves, the *Psav*‐*cneA* mutant was unable to induce an HR (Fig. 4A). Likewise, *Psav*‐*cneA* mutant was significantly affected in the ability to induce knots on olive (Fig. 4B). In fact, olive plants inoculated with the *Psav*‐*cneA* mutant showed a drastic reduction in knot overgrowth (Fig. 4C). It must be pointed out that the residual stem overgrowth seen on the *Psav*‐*cneA* mutant inoculated plants was due to the formation of cicatrisation callus as a consequence of the wounding (incisions). Moreover, we found that the *Psav*‐*cneA* mutant strain was unable to proliferate in olive plants in comparison with the *Psav* wild type (Fig. 4D).

### In the *Psav*‐*cneA *mutant expression of genes involved in type III secretion and phytohormone production are suppressed

**Figure 5:**
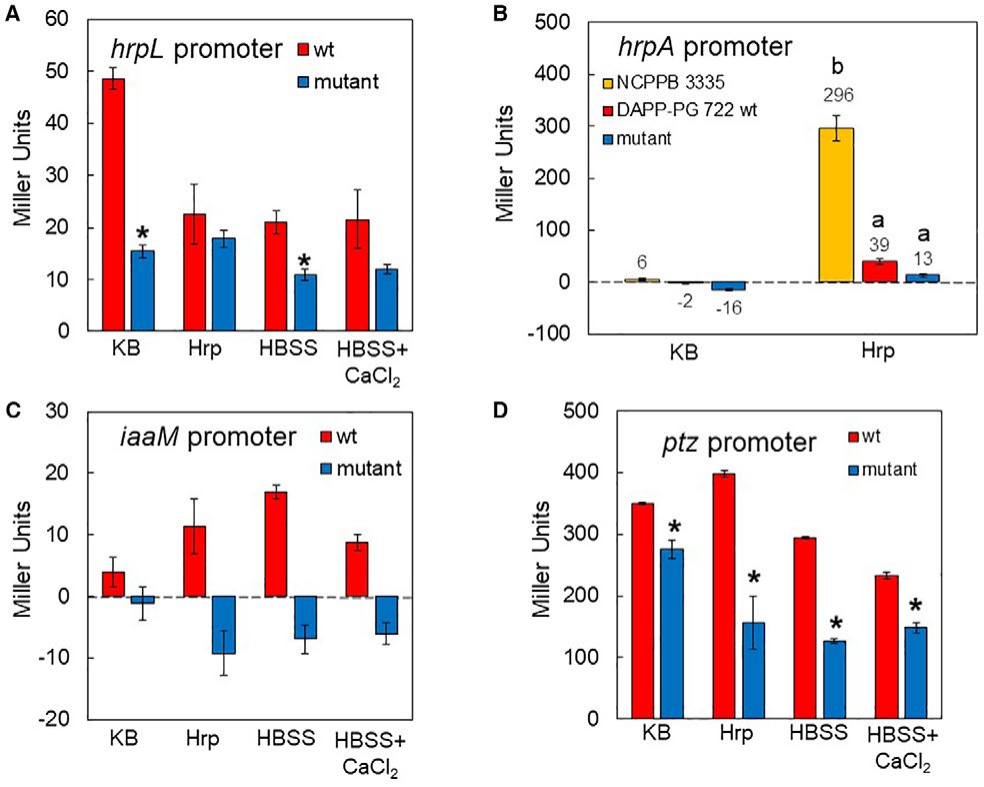
Gene expression levels of the *hrpL* (A), *hrpA* (B), *iaaM* (C), and *ptz* (D) using a promoter *LacZ* reporter system in *Pseudomonas savastanoi* pv. *savastanoi* (*Psav*) DAPP‐PG 722 (wild type [wt], red columns) and the calcium exchanger* Psav*‐*cneA* mutant (blue columns). Bacterial β‐galactosidase (LacZ) activity was measured 6 h after incubation in King’s medium B (KB), Hrp, HBSS and HBSS+CaCl_2_ media. As a negative control, *Psav* wt and *Psav*‐*cneA* mutant strains transformed with a promoterless β‐galactosidase were used. For comparison, *hrpA* promoter activity in *Psav* NCPPB 3335 strain (yellow column) was included. Each column is the mean of one experiment with three replicates ± SE. ^*^For each medium, values recorded in the *Psav*‐*cneA* mutant are statistically different (*P* < 0.05) respect to that of *Psav* wt, according to the Student’s *t*‐test. Columns capped with different letters, in Figure 5B, are significantly different (*P* < 0.05) according to the Duncan’s multiple range test.

In order to investigate the expression of genes involved in pathogenicity and virulence of *Psav,* the promoter activity of the *hrpL*, *hrpA*, *iaaM *and *ptz* genes was determined via transcriptional fusions of their gene promoters with the promoterless *lacZ* gene. Although β‐galactosidase levels associated to the *hrpL *and *hrpA* promoters were very low under the conditions tested (Fig. 5A and B), transcription from the *hrpL* promoter was significantly reduced in the *cneA* mutant grown either in KB or HBSS media (Fig. 5A). When the *Psav*‐*cneA* mutant strain was grown in Hrp medium (Huynh *et al*., 1989), the activity of the *hrpA *promoter was reduced in comparison to that obtained for wild‐type *Psav* (Fig. 5B). In addition, *hrpA *promoter activity was significantly lower in *Psav* DAPP‐PG 722 than in *Psav* NCPPB 3335 (Fig. 5B). In *Psav*‐*cneA* mutant cells, the activity of the *iaaM* promoter was also low in all media tested (Fig. 5C). Nevertheless, the activity of this promoter was significantly lower in the *cneA *mutant than in the wild‐type strains in Hrp medium, HBSS and HBSS amended with CaCl_2_. Furthermore, a significant reduction in the *ptz* promoter activity was seen in *Psav*‐*cneA* mutant compared to *Psav* wild type in all media analysed (Fig. 5D). Together, these results suggest that Na^+^/Ca^2+^ exchanger is needed for the proper expression of the tested pathogenicity and virulence genes under inducing conditions.

### *Psav‐cneA *mutant was restored by gene complementation

**Figure 6:**
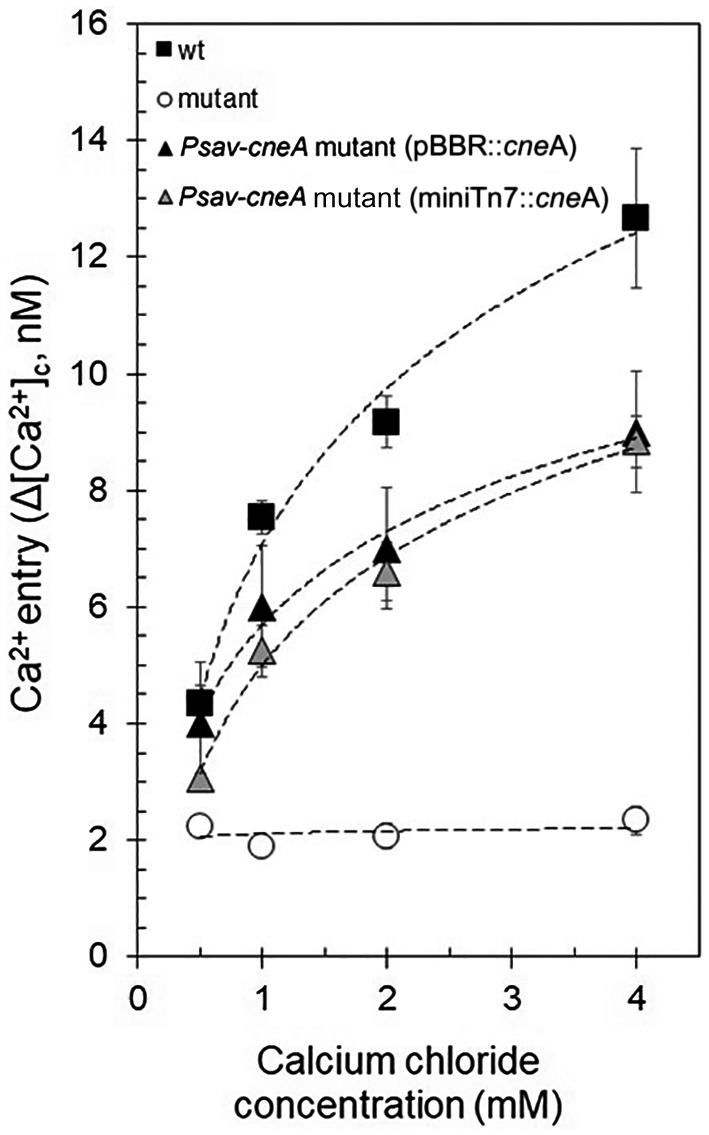
Complementation of *Psav*‐*cneA* mutant restores Ca^2+^ entry. Shown are the cytosolic Ca^2+^ levels in *Pseudomonas savastanoi* pv. *savastanoi* (*Psav*, wild type [wt], closed squares), *Psav*‐*cneA* mutant (open circles), *Psav*‐*cneA* mutant (pBBR::*cneA*) (plasmidic complementation, closed triangle) and *Psav*‐*cneA* mutant (miniTn*7*::*cneA*) (chromosomal complementation, grey triangle) cells incubated in HBSS medium alone (basal conditions) at different concentrations of extracellular calcium chloride. Each point is the mean of 10 independent experiments ± SE.

**Figure 7:**
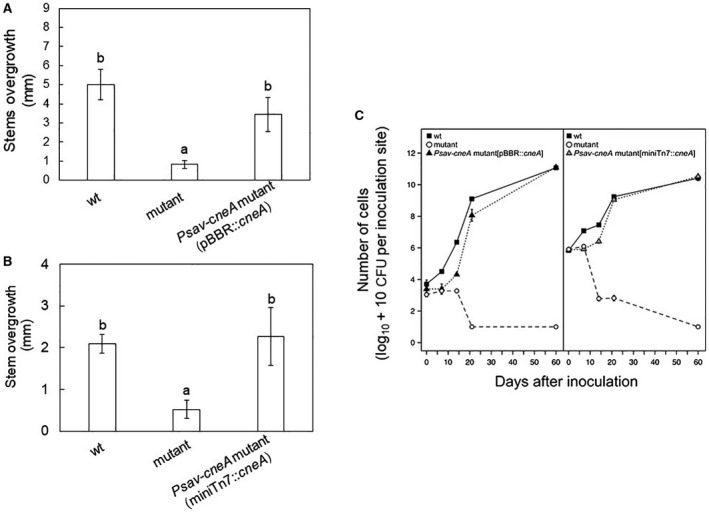
Effect of plasmidic and chromosomal complementation of the calcium exchanger mutant (*Psav*‐*cneA* mutant) on knot formation (A and B) and *in planta* population dynamics (C). (A) Knot formation, expressed as stem overgrowth observed 60 dpi, in olive (cv. Frantoio) inoculated plants with *Pseudomonas savastanoi* pv. *savastanoi* (*Psav*, wild type [wt]), *Psav*‐*cneA* mutant, and *Psav*‐*cneA* mutant (pBBR::*cneA*) (plasmidic complemented mutant). Each column represent the mean of four replicates ± S.E. Columns capped with different letter are significantly different (*P* < 0.01) according to the Duncan’s multiple range test. (B) Knot formation, expressed as stem overgrowth observed 60 dpi, in olive (cv. Frantoio) inoculated plants with *Psav *wt, *Psav*‐*cneA* mutant, and *Psav*‐*cneA* mutant (miniTn*7*::*cneA*) (chromosomal complemented mutant). Each column represent the mean of four replicates ± S.E. Columns capped with different letter are significantly different (*P* < 0.01) according to the Duncan’s multiple range test. (C) Population dynamics of *Psav* wt (closed squares), *Psav*‐*cneA* mutant (open circles), *Psav*‐*cneA* mutant (pBBR::*cneA*) (closed triangle), and *Psav*‐*cneA* mutant (miniTn*7*::*cneA*) (grey triangle) in inoculated olive (cv. Frantoio) plants. Each point is the mean of four replicates ± SE.

Complementation of the *Psav*‐*cneA* mutant was performed using both a plasmid encoding the *cneA* gene expressed from the *E. coli* *lac* promoter (*Psav*‐*cneA* mutant [pBBR::*cneA*]) or a mini‐Tn*7* transposon encoding *cneA* from its own promoter and inserted in the chromosome of the mutant strain (*Psav*‐*cneA* mutant [miniTn*7*::*cneA*]). Ca^2+^ entry into the complemented strains was restored to more than 60%, in the absence (Fig. 6) of glucose. Next, we assessed the virulence of the complemented strains on olive plants. The knot overgrowth generated by *Psav*‐*cneA* mutant (pBBR::*cneA*) was not significantly different to that of *Psav* wild type (Fig. 7A), but it was significantly higher compared to that of *Psav*‐*cneA* mutant (Fig. 7A). Also bacterial proliferation of the *Psav*‐*cneA* mutant (pBBR::*cneA*) in olive plants was comparable to the *Psav* wild type (Fig. 7B). Similar results were obtained for *Psav*‐*cneA* mutant (miniTn*7*::*cneA*) (Fig. 7C and D). This means that gene complementation restored bacterial pathogenicity and virulence on olive plants to wild‐type levels.

### Other phenotypic characteristics of the *Psav*‐*cnaA *mutant

**Figure 8:**
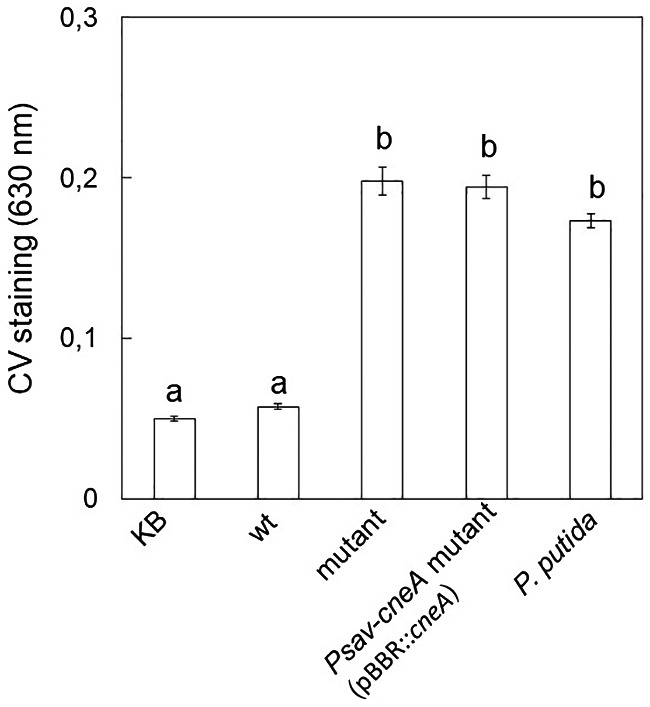
Biofilm formation measured by crystal violet (CV) staining in bacterial cells of *Pseudomonas savastanoi* pv. *savastanoi* (*Psav*) DAPP‐PG 722 (wild type [wt]), calcium exchanger* Psav* mutant (*Psav*‐*cneA* mutant), plasmidic complemented *Psav* mutant (*Psav*‐*cneA* mutant [pBBR::*cneA*]) and *Pseudomonas putida* KT2440 (positive control) grown for 48 h in static conditions. KB = King’s medium B alone. Each column is the mean of one experiment with eight replicates ± SE. Columns capped with different letters are significantly different (*P *< 0.05) according to the Duncan’s multiple range test.

In order to determine if the *Psav*‐*cneA* mutant is impaired in other phenotypic traits important for its epiphytic and endophytic lifestyles (Ramos *et al*., 2012; Rodríguez‐Moreno *et al*., 2009), several phenotypic characters were tested (Table 1). The mutant was impaired in the production of exopolysaccharides (EPSs), both in KB and LBS media, and N‐acyl homoserine lactones (AHLs), and it showed a higher swimming motility than the wild‐type strain. No difference in proteolytic activity, siderophore production and swarming motility was observed between wild type and *Psav*‐*cneA* mutant. At 24 h neither wild type nor the mutant formed biofilms under shaking or static conditions. The same results were obtained after 48 h incubation in shaking conditions. However, biofilm formation (similar to those of *Pseudomonas putida* KT2440; positive control) was detected in the *Psav*‐*cneA* mutant strain 48 h after incubation at static conditions, while no formation was detected in the wild‐type strain under these conditions (Fig. 8). Amongst the phenotypic characteristics examined, the complemented strains (*Psav*‐*cneA* mutant [pBBR::*cneA*], *Psav*‐*cneA* mutant [miniTn*7*::* cneA*]) were not able to swim as the *Psav* wild type under the conditions tested, and they only recovered partially the capacity to produce EPSs (Table 1). Other phenotypes that were not restored in the complemented strains include the induction of the HR on tobacco plants, the production of AHLs (Table 1) and the inability to form biofilms under the conditions tested (Fig. 8).

**Table 1:** Phenotypic characterization of *Pseudomonas savastanoi *pv.* savastanoi *(wild type), *Psav*‐*cneA* mutant, and two complemenation lines *Psav*‐*cneA* (pBBR::*cneA*) and *Psav*‐*cneA* mutant (miniTn*7*::*cneA*).

	Wild type	*Psav*‐*cneA*	*Psav*‐*cneA* (pBBR::*cneA*)	*Psav*‐*cneA* (miniTn*7*::*cneA*)
Hypersensitive reaction	+	−	−	−
Proteolytic activity	−	−	−	−
Siderophore production	+	+	+	+
EPS production	+	−	+/−	+/−
Swimming	−	+	−	−
Swarming	−	−	−	−
AHL production	+	−	−	−

+, positive; −, negative; +/−, weak positive.

## Discussion

Based on our data, we propose that in the early phases of the *Psav* infection and in particular when the bacterium reaches the apoplast (intercellular spaces and xylem), the abundant presence of Ca^2+^ (Stael *et al*., 2011) and the low concentration of sugars (Rico *et al*., 2009) therein permit Ca^2+^ entry into the bacterial cells via the Na^+^/Ca^2+^ exchanger cneA, which in turn induces the expression of *Psav* pathogenicity and virulence genes. Although the level of Ca^2+^ in olive apoplast has not been reported, its concentration is likely sufficient to guarantee Ca^2+^ influx in the *Psav* cells. In fact, the Ca^2+^ concentrations used in this study are consistent with those reported in plant apoplast (Hepler, 2005; Plieth and Vollbehr, 2012), which range from 10 µM to 10 mM. In addition, during the early phase of bean infection with avirulent and virulent *Pseudomonas savastanoi* pv. *phaseolicola* strains, an increase in apoplastic Ca^2+^ was documented (O'Leary *et al*., 2016). Our biochemical experiments demonstrated that Ca^2+^ entry in *Psav* cells is inhibited by glucose, fructose or sucrose. Although the concentration of these sugars in olive apoplast has not been documented, their concentrations in the apoplast of other plants is low (Preston, 2017) and decrease during the early phase of a bacterial infection (O'Leary *et al*., 2016). Even though the level of these sugars in the olive apoplast should attenuate Ca^2+^ entry, we have to consider that minimal changes in cytosolic Ca^2+^ concentration can modulate gene expression (Borowiec *et al*., 2014; Domínguez, 2004). We therefore, hypothesize that a sugar starvation status can facilitate the entry of Ca^2+^ inside *Psav* cells. A high degree of starvation already occurs during the epiphytic phase of *Psav*, which is able to live on olive leaf surfaces exploiting the poor nutrients there present (Ramos *et al*., 2012). We cannot exclude that in this ecological niche, Ca^2+^ present in water and stored in EPSs enters into the *Psav* cells to regulate important processes that control in the epiphytic life style. The starvation experience during the epiphytic phase of the life cycle, is mitigated as soon as the bacteria enter the apoplast; however, a limited amount of starvation is always present in the apoplast that is considered a nutrient‐limited environment (Rico *et al*., 2009), supporting the existence of certain starvation conditions also in this niche.

The importance of Ca^2+^ for the virulence of a phytopathogenic bacterium was recently reported by Fishman *et al*. (2018), who characterized a two‐component system of *P. syringae* pv. *tomato* DC3000 that is responsive to Ca^2+^ and necessary for virulence of this bacterium. Through the use of a Na^+^/Ca^2+^ exchanger mutant, we now identify for a related bacterium, *Psav,* an exchanger that is essential for Ca^2+^ influx. In corroboration, we demonstrate at the biochemical and pharmacological level that Ca^2+^ enters *Psav* bacterial cells via this Na^+^/Ca^2+^ exchanger that belongs to the ChaA antiporter superfamily (Shijuku *et al*., 2002). Using a genomic knockout mutant and genetic complementation, we have shown that this exchanger is essential for *Psav *virulence on olive plants, as the mutant failed to induce knots. We also demonstrate that Ca^2+^ entry stimulates the expression of both pathogenicity (*hrpL*, *hrpA* and* iaaM*) and virulence (*ptz*) genes again confirming that Ca^2+ ^is an important host signal that is perceived by the bacterium. We find that the Ca^2+^ influx reaches its maximum levels when the energy supply is limiting. In fact, the presence of glucose, fructose, sucrose or ATP inhibited calcium entry entirely.

Thus far, *Psav* virulence was largely linked to the bacterial secretion of the phytohormones IAA and cytokinins at the site of infection, which stimulates olive cell activity to produce new tissue and gives rise to knot development (Glass and Kosuge, 1988; Powell and Morris, 1986; Rodríguez‐Moreno *et al*., 2008; Quesada *et al*., 2012; Surico *et al*., 1985; Temsah *et al*., 2008). Our results demonstrate that the presence of L‐tryptophan or IAA does not alter Ca^2+^ entry into the *Psav* cells, suggesting that there is no feedback regulation by the auxin pathway during infection. Our data imply that Ca^2+^ entry regulates other virulence factors in *Psav* DAPP‐PG 722 such as EPS, AHLs and biofilm production as well as swimming motility. In the *Psav*‐*cneA* mutant lack of Ca^2+^ entry affects specifically EPS and AHLs production. In the marine bacteria *Pseudoalteromonas* sp., in *Pseudomonas putida* and in *Pseudomonas aeruginosa*, Ca^2+^ influences the production of the extracellular matrix and adhesion to seeds (Espinosa‐Urgel *et al*., 2000; Patrauchan *et al*., 2005; Sarkisova *et al*., 2005). In *Xylella fastidiosa* Ca^2+^ did not directly affect EPS production while being involved in the regulation of biofilm formation, cell surface attachment and twitching motility (Cruz *et al*., 2012, 2014, 2012, 2014; Parker Jennifer *et al*., 2016). The opposite effect was observed in *Psav*‐*cneA* mutant, *i.e.* enhanced biofilm formation after 48 h in static conditions and increased swimming motility. The *Psav‐cneA* mutant strain fails to elicit disease symptoms in its host and HR on its non‐host (tobacco) probably due to the suppression of type III secretion system. Here, in fact, it was noted that β‐galactosidase activity associated to *hrpL *and *hrpA* promoters was statistically reduced in the *Psav*‐*cneA* mutant. Wei *et al*. (2000) reported that HrpA may have a positive regulatory effect on *hrpRS* and *hrpL* genes expression in *P*. *syringae* pv. *tomato*. Based on the results obtained in this study, it can be argued that Ca^2+^ positively controls expression of the genes for the type III secretion system.

Our results obtained with the deletion mutant were confirmed by plasmid and chromosomal mutant complementation, except for restoration of the HR. AHLs production and biofilm formation were also not recovered. This, may be due to the different expression levels of *cneA* in the complemented strain compared to the wild type (in the complementation line, *Psav*‐*cneA* mutant (pBBR::*cneA*), the expression was driven by the lac promoter). However, it should be emphasized that complementation of the *Psav*‐*cneA* mutation restored both Ca^2+^ entry and pathogenicity in olive plants.

## Experimental Procedures

### Bacterial strains, plasmids and growth conditions

Bacterial strains and plasmids used in this study are listed in Table 2. Bacterial strains were grown at 27 °C in Luria‐Bertani (LB) medium (Miller, 1972), King’s B (KB) medium (King *et al*., 1954) or Nutrient Agar (NA). *Escherichia coli* was grown at 37 °C in LB broth. *Chromobacterium* *violaceum* strain CVO26 (McClean *et al*., 1997), used as AHL bacterial biosensor for AHL detection, was grown at 30 °C. Antibiotics were added, when required, at the following final concentrations: ampicillin 100 μg/mL, nitrofurantoin (Nitrof) 100 μg/mL, kanamycin (Km) 100 μg/mL and gentamicin (Gm) 10 μg/mL.

**Table 2:** Bacterial strains, plasmids and primers used in this study.

Strains	Relevant characteristics*	References
*Pseudomonas savastanoi* pv. savastanoi (*Psav*)		
DAPP‐PG 722 (wild type)	Olive knot (Italy)	Moretti *et al.* (2014)
*Psav*‐*cneA* mutant	Interruption *cneA* mutant (Nitrof^R^ – Km^R^) of *Psav *DAPP‐PG 722	This study
Plasmid complemented strain	*Psav*‐*cneA* mutant (pBBR::*cneA*)	″
Chromosomal complemented strain	*Psav*‐*cneA* mutant (miniTn*7*::*cneA*)	″
*Escherichia coli*		
DH5α	*F ‐, *ϕ80dlacZ *M15, (lacZYA‐argF) U169, deoR, recA1, endA, hsdR17 (rk ‐ mk ‐), phoA, supE44, thi‐1, gyrA96, relA1*	Hanahan (1983)
Plasmids:		
pKNOCK‐Km	Conjugative suicide vector; Km^R^	Alexeyev (1999)
pKNOCK‐cneA	Internal PCR EcoRV *cneA* fragment of *Psav* cloned in pKNOCK‐Km	This study
pBBR MCS‐5	Broad‐host‐range cloning vector; Gm^R^	Kovach *et al*. (1995)
pBBR MCS‐5‐cneA	pBBRMCS5 with 1.1 kb *Xho*I ‐ *Spe*I fragment containing the *cneA* gene of *Psav*	This study
pGEM^®^‐T Easy vector	Cloning vector; Amp^R^	Promega, Fitchburg, WI, USA
pUC18R6KT‐miniTn*7*BB‐Gm	Cloning vector; Gm^R^	Caballero and Govantes (2011)
pUC18R6KT‐miniTn*7*BB‐cneA‐Gm	pUC18R6KT‐miniTn*7*BB‐Gm containing the *cneA* gene of *Psav*	This study
Primers:		
cneA For	5′‐GGCGAGCAGTCCTATAACGAT‐3′	This study
cneA Rev	5′‐ACACCGATGACCAATGTGACA‐3′	″
cneA compl 1	5′‐CTCGAGAGGAGGATGGGCGCTTTGCTCAAGC‐3′	″
cneA compl 2	5′‐CCTAGGCTAAAGCCCCAGACACGAG‐3′	″
PromAP_Fw	5′‐CAGAAGCTGAATCGTGAAAA‐3′	″
AP_Rev	5′‐TGGGAGCGATAGGCAATA‐3′	″
glmS_savastanoi	5′‐AACCTGGCGAAGTCGGTGAC‐3′	″
Tn7Rev	5′‐CAGCATAACTGGACTGATTTCAG‐3′	″
Primers for β‐galactosidase activity:		
iaaM For	5′‐ACTCATGGAGATCTGAAAATCTGGTGCTGATGC‐3′	Aragόn *et al*. (2014)
iaaM Rev	5′‐ACTCATGGGGTACCCTATGCCTCCCGTCATTTC‐3′	″
ptz For	5′‐ACTCATGGAGATCTATGCCGACTTGAGTAATCGG‐3′	″
ptz Rev	5′‐ACTCATGGGGTACCTCCGGTACAAGTAGCACCC‐3′	″
hrpA For	5′‐GACGAATTCGAAAAGGCCCTGATTCAACA‐3′	″
hrpA Rev	5′‐TACGGATCCGACCCGCGTTAGTCAGAGAA‐3′	″
hrpL For	5′‐CCCGAATTCGGCGACGATTTCATAGGAC‐3′	″
hrpL Rev	5′‐CCCGGATCCGTTGGAAACATGGGCTTAC‐3′	″

*Nitrof, nitrofurantoin; Km, kanamycin; Gm, gentamycin; Amp, ampicillin.

### Recombinant DNA techniques

DNA digestions with restriction enzymes (XhoI, SpeI and EcoRI), agarose gel electrophoresis, DNA fragment purification, ligation with T4 ligase, end filling using the Klenow enzyme and *E. coli* transformation were performed as described by Sambrock *et al*. (1989). Plasmids were purified using the GenElute™ Plasmid Miniprep Kit (Sigma‐Aldrich, MO, Saint Louis, USA). The genomic DNA was extracted with the GenElute Bacterial Genomic DNA Kit (Sigma‐Aldrich, MO, Saint Louis, USA). Triparental mating between *E. coli* and *Psav* DAPP‐PG 722 was performed using a helper *E. coli* strain carrying plasmid pRK2013 (Figurski and Helinski, 1979).

### Determination of the cytosolic Ca^2+^ levels

Cytosolic Ca^2+^ levels were determined using a fluorimetric method, which employed the fluorescent probe Fura 2‐AM (Fura 2‐acetoxy methyl ester; Sigma‐Aldrich, MO, Saint Louis, USA). Approximately 5 ×  10^6^ cells of *Psav* DAPP‐PG 722 grown at 27 ± 1°C for 16 h in LB broth to a stationary phase, were suspended in 0.12 M Tris (pH 7.8) and 2 mM EGTA. At 200 s after incubation at 25 °C, 2 mM CaCl_2_ was added to stop the EGTA effect as reported by Grynkiewicz *et al*. (1985). Then the cells were incubated for 2 h in basal condition *i.e.* HBSS buffer (140 mM NaCl, 5.3 mM KCl, 25 mM HEPES, pH 7.4) supplemented with 2 mM Fura 2‐AM (dissolved in DMSO) or in HBSS buffer supplemented with 2 mM Fura 2‐AM and different carbon sources (glucose, fructose or sucrose, 5 mM) or ATP 50 µM. The fluorescence intensities of Fura 2‐AM (Ex. = 335 nm, Em. = 505 nm) were monitored with a spectrofluorophotometer (Perkin‐Elmer, Waltham, Massachusetts, USA). The cytosolic Ca^2+^ concentration was calculated following the formula reported by Grynkiewicz *et al*. (1985).

### Phylogenetic analysis of the Na^+^/Ca^2+^ exchanger gene

A comparative phylogenetic analysis of the nucleotide sequences of the *cneA* gene coding for the Na^+^/Ca^2+^ exchanger was performed using the Geneious resource (Kearse *et al*., 2012). Blast searches were used to retrieve the close homologs of the *cneA* gene from different *Pseudomonas* species. Phylogenetic and molecular evolutionary analysis was conducted using MEGA 7 (Kumar *et al*., 2016) and the maximum likelihood method. Clade stability was assessed by 1000 bootstrap replications.

### Construction of a *P. savastanoi* pv. *savastanoi* knockout mutant of the Na^+^/Ca^2+^ exchanger gene *cneA*

A genomic null mutant of the Na^+^/Ca^2+^ exchanger gene (referred to as *cneA* gene) was created as follows. An internal 305 bp fragment of the *cneA* gene was amplified from *Psav* DAPP‐PG 722 genomic DNA using the primers cneA For and cneA Rev (Table 2). The amplified PCR product was cloned in plasmid pKNOCK‐Km (Alexeyev, 1999), generating pKNOCK‐cneA (Table 2). A *Psav*‐*cneA* knockout mutant (Table 2) was generated by homologous recombination (Alexeyev, 1999) after transformation of pKNOCK‐cneA in *Psav* DAPP‐PG 722 as a suicide delivery system. Transformants were selected on KB‐Nitrof + Km plates. Interruption of *cneA* was verified by PCR using primers specific to the pKNOCK‐Km vector and to the genomic DNA sequences upstream and downstream of the targeted gene. The amplicons were sequenced at Macrogen Europe (Amsterdam, Netherlands; http://www.macrogen.com).

### Plasmid and chromosomal complementation of *Psav‐cneA* mutant

Complementation of* Psav*‐*cneA* mutant with a plasmid encoding the *cneA* gene was performed as follows. The complete sequence of the *cneA* open reading frame (ORF) with its ribosome binding site was amplified from *Psav* DAPP‐PG 722 genomic DNA using primers cneA compl 1 and cneA compl 2 (Table 2) and Q5^®^High‐Fidelity DNA Polymerase (New England Biolabs, Hitchin, UK). The amplified fragment was purified from an agarose gel using the EuroGOLD Gel Extraction Kit (EuroClone, Milan, Italy) following the instructions of the manufacturer. After A‐tailing (Promega, Fitchburg, WI, USA), the fragments were cloned in pGEM‐T Easy vector (Promega, Fitchburg, WI, USA) and sequenced at Macrogen Europe. Having verified the correctness of the sequence, the *cneA* ORF was excised from pGEM‐T Easy Vector using *XhoI* and *SpeI* and cloned in the corresponding sites of the plasmid pBBR MCS‐5. The resulting plasmid (pBBR MCS‐5‐cneA; Table 2) was purified using the GenElute Plasmid Miniprep Kit (Sigma‐Aldrich, MO, Saint Louis, USA) and transformed in *Psav‐cneA* by electroporation, generating *Psav*‐*cneA* mutant (pBBR::*cneA*) (Table 2).

Chromosomal complementation of the *Psav‐cneA* mutant was performed using the Tn*7* transposon vector pUC18R6KT‐miniTn*7*BB‐Gm. The complete ORF of the *cneA* gene, including its own promoter, was amplified from *Psav* DAPP‐PG 722 chromosomal DNA using the Expand High Fidelity PCR System (Roche, Mannheim, Germany) and the primers PromAP_Fw and AP_Rev (Table 2). The amplified DNA fragment was cloned in the pGEM‐T Easy Vector (Promega, WI, Fitchburg, USA) and sequenced at GATC Biotech (Konstanz, Germany). Once verified the correctness of the sequence, the *cneA* gene was excised from pGEM using EcoRI and cloned in the corresponding site of the plasmid pUC18R6KT‐miniTn*7*BB‐Gm, yielding pUC18R6KT‐miniTn*7*BB‐cneA‐Gm (Table 2) that was electroporated in *Psav‐cneA*. Selection of the transconjugants in KB‐Gm plates yielded the complemented strain *Psav*‐*cneA* mutant (miniTn*7*::*cneA*) (Table 2). Insertion of the Tn*7* transposon into the correct site was verified using the primers GlmS_savastanoi (hybridizing at the 3′ of the *glmS* gene) and the Tn7Rev primer (hybridizing at the Tn7R end of the integrated plasmid) (Table 2). Only in the case of integration, a 165 bp fragment was amplified.

### Phenotypic characterizations of the* Psav‐cneA *mutant and its complemented strains

*In vitro* bacterial growth dynamics of wild‐type* Psav* and the *Psav*‐*cneA *mutant strains were carried out in KB liquid medium at 27 °C. Bacterial growth was spectrophotometrically followed every hour for 24 h at OD_660_ and through colony counts at 4, 8, 20, 24 and 28 h post‐incubation (hpi). For each bacterial strains, the relationship between the number of cells (log_10_ transformed) and the hpi was investigated by means of a second‐order polynomial model. Likelihood ratio test was used to assess the differences between wild type and *Psav*‐*cneA* mutant strains under R statistical environment (R Core Team, 2018).

The HR assay was carried out in *Nicotiana tabacum* (cv. Havana 425) plants. To prepare the inoculum, the strains were grown in NA at 27 °C for 24 h, resuspended in sterile deionized water and spectrophotometrically adjusted to 10^8^ CFU/mL. About 10 µL of the bacterial suspensions or water (control) was infiltrated into the mesophyll of tobacco leaves using a needleless syringe. The appearance of the HR was scored at 24 hpi.

Proteolytic activity, swarming and swimming were determined as reported by Huber *et al*. (2001). Qualitative analysis of EPSs was tested on KB and LB solid medium amended with 5% of sucrose (LBS). Single colonies, previously obtained from NA plates, were streaked on KB and LBS and then grown at 28 ºC for 48 h. Colonies producing EPSs showed a fluidal, mucoid appearance. Production of AHLs was performed in T‐streak analysis as described by Piper *et al*. (1993) using the *C. violaceum* CVO26 as AHL biosensor. To measure biofilm formation, overnight cultures of *Psav* DAPP‐PG 722, *Psav*‐*cneA* mutant and *Psav*‐*cneA* mutant (pBBR::*cneA*) grown in KB broth, were diluted to OD_600nm_ = 0.1 and loaded in a 96‐well plate (150 µL per well, eight wells per strain). Plates were incubated under static or shaking conditions and biofilm formation was quantified by measurement of the A_595_ after 24 h and 48 h after crystal violet staining (O'Toole and Kolter, 1998). *Pseudomonas putida *KT2240 was included as a positive control for biofilm formation and cell‐free KB as negative control.

### Pathogenicity test on olive plants

Disease severity and bacterial growth were tested in 1‐year‐old olive (cv. Frantoio) plants inoculated with the strains *Psav* DAPP‐PG 722, *Psav*‐*cneA* mutant*, Psav*‐*cneA* mutant (pBBR::*cneA*) and *Psav*‐*cneA* mutant (miniTn*7*::* cneA*) (Table 2). To prepare the inoculum, bacteria were grown on NA at 27 °C for 48 h, resuspended in sterile deionized water and adjusted spectrophotometrically to approximately 1 ×  10^8 ^CFU/mL^−1^. Also, 20 μL of bacterial suspension or water (control plant) was placed in wounds (five per plant) made in the bark of olive plants using a sterile scalpel as previously described (Moretti *et al*., 2008). Wounds in the inoculated and control plants were protected with parafilm (American National Can, IL, Chicago, USA) until the developing knots break it (14 to 21 days). Plants were maintained in transparent polycarbonate boxes to reach high RH values (90%–100%) and kept in a growth chamber at 22 °C to 24 °C with illumination at 70 μE/m^‐2^s^‐1^ and 12 h light period. The *Psav *population density was calculated at 0, 7, 14, 21 and 60 dpi by serial dilution of the bacterial suspension obtained from inoculated sites excised and homogenized by mechanical disruption and plated in NA medium. Colony counts were calculated 24 h and 48 h after incubation at 27 °C. The disease severity was recorded at 60 dpi by determining the knot volume, by measuring the length, width and depth of every knot with a Vernier caliper (Moretti *et al*., 2008). Four plant replicates were included in each of the two *in planta* experiments performed.

### Transcriptional analysis of* Psav* pathogenicity and virulence genes

To verify whether Ca^2+^ entry promotes the expression of pathogenicity (*hrpL*, *hrpA* and *iaaM*) and virulence (*ptz*) genes of* Psav*, transcriptional fusions of their promoters were constructed with *LacZ *reporter gene. For amplification of the *iaaM* and *ptz* promoters, the regions upstream of the *iaaM* and *ptz* ORFs (477 bp and 373 bp, respectively) were amplified by PCR using primers iaaM For, iaaM Rev, ptz For and ptz Rev (Table 2). Amplicons were cloned into pMP220 in order to obtain promoter fusions to *lacZ*. The resulting plasmids and those encoding the *hrpL* and *hrpA* promoters fused to *lacZ* (Aragόn *et al*., 2014) were transferred by conjugation into both wild‐type *Psav* DAPP‐PG 722 and its *cneA* mutant. Cells carrying the plasmids grown overnight in KB media were diluted in the same media and incubated at 28 ºC to OD_660_ of 0.5 (time = 0). The cultures were harvested by centrifugation, washed twice with 10 mM MgCl_2_ and the cells were transferred to Hrp medium (Huynh *et al*., 1989), HBSS and HBSS amended with CaCl_2_. The cultures were adjusted to OD_660_ of 0.5 and incubated for 6 h at 28 ºC. β‐galactosidase enzymatic activity was measured using the methods developed (Miller, 1972) and modified previously (Maloy, 1990). *Psav* DAPP‐PG 722 and its *cneA* mutant transformed with pMP220 (encoding a promoterless *lacZ*) were used as negative controls. To determine the activity associated exclusively to the promoter fusions to *lacZ*, the background activity detected in the control strains was subtracted from those obtained for each of their corresponding transformants.

## Compliance with Ethical Standards

Conflict of interest: The authors have declared that no conflict of interest exists.

Research Involving Human Participants and/or Animals: This article does not contain any studies with human participants or animals (vertebrates) performed by any of the authors.

Informed consent: Informed consent was obtained from all individual participants included in the study.

## Supporting information


**Fig. S1** Maximum likelihood tree based on the nucleotide sequence of the *cneA* gene showing the phylogenetic relation within the *P. syringae* complex. Phylogroup (PG) designations are indicated on the appropriate branches. Numbers at branching points are bootstrap percentages based on 1000 replications. *Psy *= *Pseudomonas syringae*; *Psav* = *Pseudomonas savastanoi*; *Pca *= *Pseudomonas cannabina* and *P *= *Pseudomonas*.

**Fig. S2** *In vitro* growth on KB medium of *Pseudomonas savastanoi* pv. *savastanoi* (*Psav*) DAPP PG 722 (wild type [wt]) and the calcium exchanger* Psav* mutant (*Psav* *cneA* mutant). Number of cells (mean ± SE) and fitted polynomial models of wt (closed squares, solid line; fitted model: y = −0.004x^2^ = 0.359x = 3.876) and *Psav* *cneA* mutant (open circles, dashed line; fitted model: y = −0.005x^2^ = 0.410x = 3.664). Standard error bars are not visible in the plot as their values are smaller than the dimensions of the closed squares and open circles.
