# Supplementary figures and images for: A Na+/Ca2+ exchanger of the olive pathogen Pseudomonas savastanoi pv. savastanoi is critical for its virulence
Source: Mol Plant Pathol. 2019 Mar 26;20(5):716–30. doi: 10.1111/mpp.12787 (PMC6637891; doi:10.1111/mpp.12787)

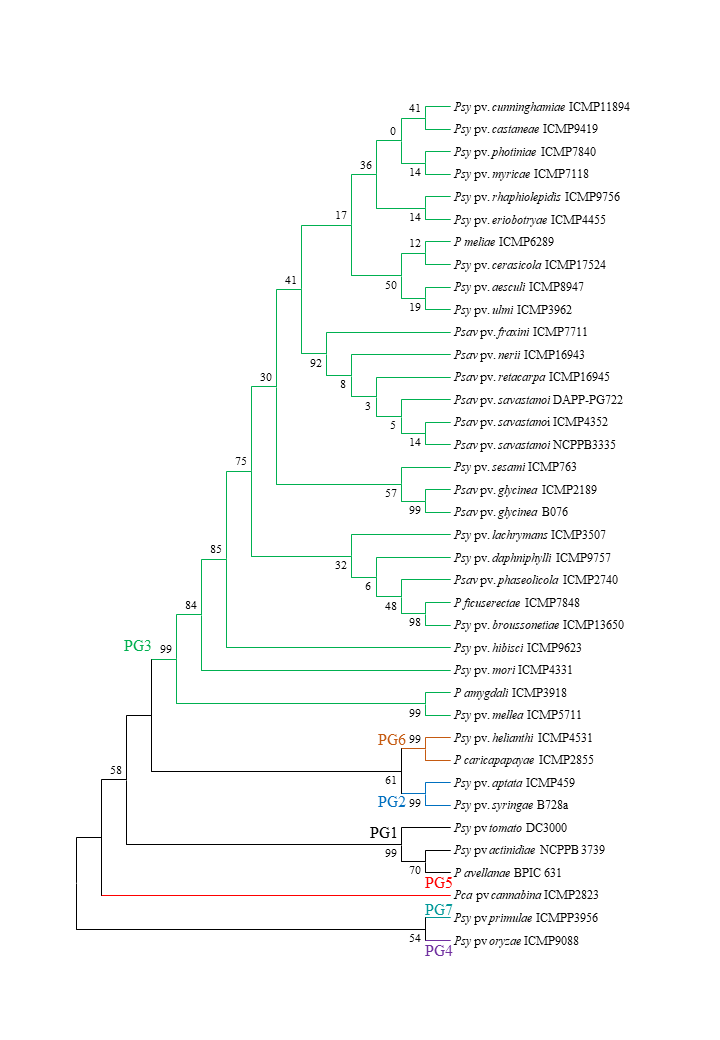

Supplement: Supplementary file 1 — Fig. S1 Maximum likelihood tree based on the nucleotide sequence of the cneA gene showing the phylogenetic relation within the P. syringae complex. Phylogroup (PG) designations are indicated on the appropriate branches. Numbers at branching points are bootstrap percentages based on 1000 replications. Psy = Pseudomonas syringae; Psav = Pseudomonas savastanoi; Pca = Pseudomonas cannabina and P = Pseudomonas. [file MPP-20-716-s001.tif]

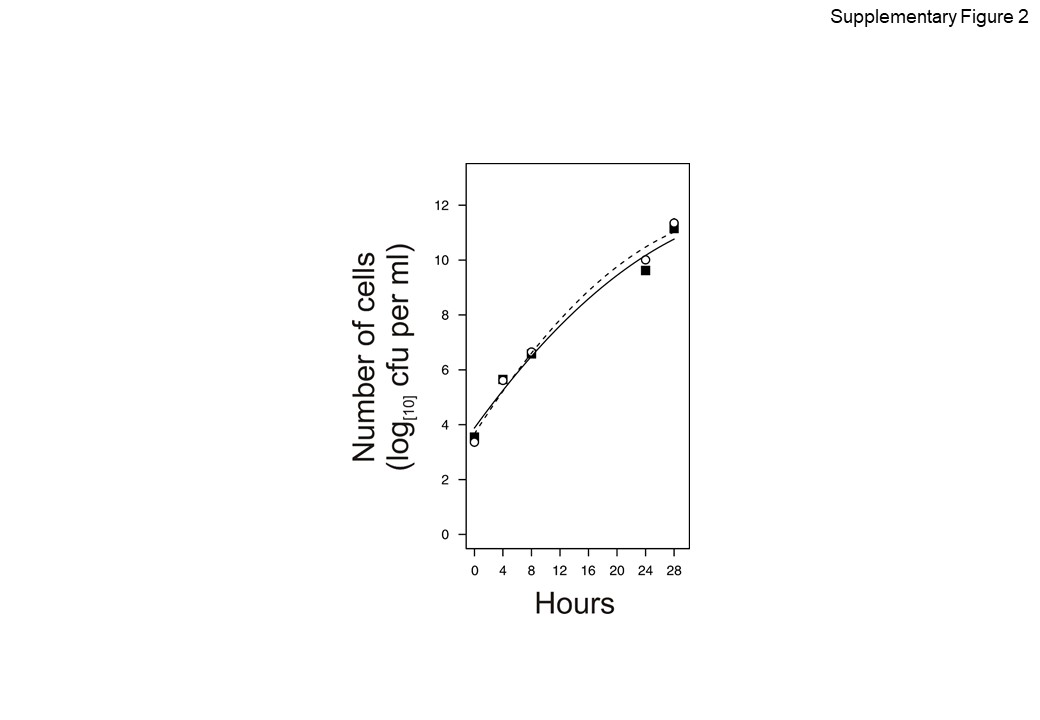

Supplement: Supplementary file 2 — Fig. S2 In vitro growth on KB medium of Pseudomonas savastanoi pv. savastanoi (Psav) DAPP PG 722 (wild type [wt]) and the calcium exchanger Psav mutant (Psav cneA mutant). Number of cells (mean ± SE) and fitted polynomial models of wt (closed squares, solid line; fitted model: y = −0.004x2 = 0.359x = 3.876) and Psav cneA mutant (open circles, dashed line; fitted model: y = −0.005x2 = 0.410x = 3.664). Standard error bars are not visible in the plot as their values are smaller than the dimensions of the closed squares and open circles. [file MPP-20-716-s002.jpg]
